# Chromatin Structure Regulates Gene Conversion

**DOI:** 10.1371/journal.pbio.0050246

**Published:** 2007-09-18

**Authors:** W. Jason Cummings, Munehisa Yabuki, Ellen C Ordinario, David W Bednarski, Simon Quay, Nancy Maizels

**Affiliations:** 1 Department of Immunology, University of Washington School of Medicine, Seattle, Washington, United States of America; 2 Department of Biochemistry, University of Washington School of Medicine, Seattle, Washington, United States of America; Brandeis University, United States of America

## Abstract

Homology-directed repair is a powerful mechanism for maintaining and altering genomic structure. We asked how chromatin structure contributes to the use of homologous sequences as donors for repair using the chicken B cell line DT40 as a model. In DT40, immunoglobulin genes undergo regulated sequence diversification by gene conversion templated by pseudogene donors. We found that the immunoglobulin Vλ pseudogene array is characterized by histone modifications associated with active chromatin. We directly demonstrated the importance of chromatin structure for gene conversion, using a regulatable experimental system in which the heterochromatin protein HP1 (Drosophila melanogaster Su[var]205), expressed as a fusion to Escherichia coli lactose repressor, is tethered to polymerized lactose operators integrated within the pseudo-Vλ donor array. Tethered HP1 diminished histone acetylation within the pseudo-Vλ array, and altered the outcome of Vλ diversification, so that nontemplated mutations rather than templated mutations predominated. Thus, chromatin structure regulates homology-directed repair. These results suggest that histone modifications may contribute to maintaining genomic stability by preventing recombination between repetitive sequences.

## Author Summary

Homologous recombination promotes genetic exchange between regions containing identical or highly related sequences. This is useful in repairing damaged DNA, or in reassorting genes in meiosis, but uncontrolled homologous recombination can create genomic instability. Chromosomes are made up of a complex of DNA and protein, called chromatin. DNA within chromatin is packed tightly in order to fit the entire genome inside a cell; but chromatin structure may become relaxed to allow access to enzymes that regulate gene expression, transcribe genes into mesenger RNA, or carry out gene replication. We asked if chromatin packing regulates homologous recombination. To do this, we tethered a factor associated with compact chromatin, called HP1, adjacent to an immunoglobulin gene locus at which homologous recombination occurs constitutively, in order to produce a diverse repertoire of antibodies. We found that the compact, repressive chromatin structure produced by HP1 prevents homologous recombination. This finding suggests that regulated changes in chromatin structure may contribute to maintaining genomic stability by preventing recombination between repetitive sequences.

## Abstract 3

Much of the chromosome is tightly packed (heterochromatic) and not transcribed. Here, the authors show that tight packing has another effect: it prevents recombination between homologous sequences.

## Introduction

Homologous recombination provides a pathway for restoring or altering DNA sequence and structure [1–7]. Homologous recombination can recreate the original DNA sequence at a DNA break, and predominates in S/G2 phases of cell cycle, when sister chromatids can serve as donors for faithful repair [8,9]. Homologous recombination can also have a mutagenic outcome by promoting recombination between nonallelic repeated sequences, leading to genomic instability, or by templating repair from a homolog rather than a sister chromatid, leading to loss of heterozygosity (LOH). In a living cell, multiple pathways compete to repair the same kinds of damage. For example, double-strand breaks (DSBs) can be repaired by nonhomologous end-joining, which may be accompanied by sequence loss or translocation [5,10,11]. Nicks can be efficiently repaired in situ, or by short- or long-patch repair pathways that use the complementary strand as a template [12,13].

Chromatin structure plays an important role in repair at the site of DNA damage. A critical signal for DSB repair is C-terminal phosphorylation of the variant histone H2AX by ATM and ATR, to generate γ-H2AX [14–16]. γ-H2AX is recruited to the break and extends over a large region surrounding the break site, creating a boundary of modified chromatin, and recruits the cohesin complex to sites of damage, to promote DSB repair using the sister chromatid as a template [17,18]. γ-H2AX alerts DNA damage checkpoints, and is recognized by histone acetyltransferases and chromatin remodeling complexes [19–22]. Changes in chromatin structure also facilitate synapsis of severed DNA ends for nonhomologous end-joining [23].

Homologous recombination involves two DNA molecules, the recipient, which is the site of the DNA lesion, and the donor. Two lines of evidence suggest that donor chromatin structure may contribute to homologous recombination. At the yeast mating type locus, changes in histone acetylation occur at the donor locus that are distinct from those at or near the DNA break [24]. In human cells, transcription of a donor promotes its use in gene conversion [25]. However, the role of donor chromatin structure in regulating recombination has not been directly tested in vertebrate cells.

**Figure 1:**
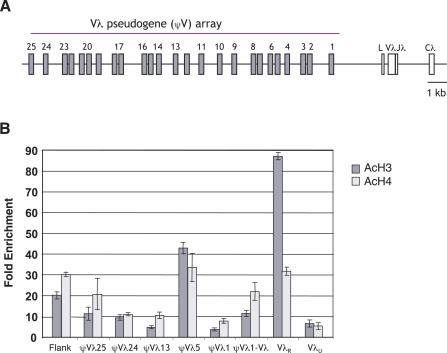
Chromatin Modification at the DT40 Igλ Locus (A) Schematic diagram of the rearranged chicken Igλ locus, showing the 25 ψVλ regions and the rearranged Vλ_R_ gene. Cλ, constant region; L, leader; VλJλ, variable region. (B) Summary of a representative chromatin immunoprecipitation experiment, assaying N-terminal acetylation of histones H3 and H4 (AcH3 and AcH4). Sites interrogated were as follows: a region approximately 1 kb upstream of the ψVλ array (flank), ψVλ25, ψVλ24, ψVλ13, ψVλ5, ψVλ1, the region between ψVλ1 and Vλ, and the rearranged Vλ_R_ and unrearranged Vλ_U_ alleles. See Materials and Methods for details. Bars indicate standard deviation.

Immunoglobulin (Ig) gene diversification in chicken B cells provides a powerful model for studying homologous recombination. Chickens have a limited number of functional heavy and light chain variable (V) regions, which undergo V(D)J recombination early in B cell development [26,27]. The rearranged V genes then undergo sequence diversification by gene conversion, using an array of homeologous upstream pseudo-V (ψV) regions as donors (Figure 1A). The ψV regions are nonfunctional, as they lack promoters and cannot be transcribed. The mechanism of Ig gene conversion is readily studied in the DT40 cell line, which derives from a bursal lymphoma and constitutively diversifies its Ig heavy and Ig light chain (Igλ) genes by gene conversion [28–30]. DT40 also supports very high levels of homologous gene targeting, thought to reflect elevated expression or activity of factors that promote recombinational repair [31–33].

Ig gene conversion in DT40 depends upon ubiquitous and conserved factors. The substrate for repair is a nick produced by successive action of three factors. The B-cell-specific enzyme activation-induced deaminase (AID) [34–37] deaminates cytosine to uracil in transcribed and targeted genes [38–41]; uracil DNA glycosylase excises the uracil produced by AID to generate an abasic site [42–46]; and the MRE11 abasic lyase, functioning within the MRE11/RAD50/NBS1 complex, nicks at the abasic site [47,48]. Strand transfer and new DNA synthesis are carried out by ubiquitous DNA repair factors including the RAD51 paralogs, BRCA2, FANCC, FANCD2, and polη [49–55]. Deficiencies in some of these factors, particularly the RAD51 paralogs, or targeted deletion of some or all of the ψV donors [56], impair gene conversion and can contribute to a shift in the processing of AID-initiated breaks so that templated repair is accompanied or even supplanted by nontemplated mutagenesis. ψV regions preferentially used as donors are in opposite orientation to the functional V region, suggesting that local chromosomal architecture may guide templated repair [57]. However, nothing is known about how epigenetic features of the donors affect recombination.

To understand how donor chromatin structure affects gene conversion in particular, and homologous recombination more generally, we characterized and experimentally manipulated chromatin structure at the Igλ locus in the chicken B cell line DT40. We found that the ψVλ donors contain acetylated histones, consistent with an open chromatin structure. To test whether this reflects requirements of gene conversion, we tethered HP1 (Drosophila melanogaster Su[var]205) to the ψVλ array in a DT40 derivative in which polymerized lactose operator (PolyLacO) has been inserted into that chromosomal region. HP1 is known to promote heterochromatic gene silencing [58–60]. Tethered HP1 caused a local transition of the donor sequences from an open to a nonpermissive state, and a switch from templated to nontemplated diversification, evident as point mutations. These observations demonstrate that permissive chromatin structure at the donor is a key regulator of gene conversion, and that nonpermissive chromatin structure can prevent homologous recombination and result in point mutagenesis. These results have implications for our understanding of homologous recombination and of the mechanisms that promote LOH, leading to tumorigenesis and nonallelic recombination between repeats. These results should also inform design of donor constructs for targeted gene therapy.

## Results

### Permissive Chromatin Structure at Vλ and ψVλ Donor Templates

In DT40 B cells, the Vλ gene is rearranged and expressed at one Igλ allele, but it is unrearranged at the other allele. We characterized chromatin structure at the rearranged (Vλ_R_) and unrearranged (Vλ_U_) alleles and the ψVλ array by chromatin immunoprecipitation (ChIP). ChIP was carried out with antibodies specific for lysine acetylation at the N-termini of histones H3 and H4. Recovered DNA was amplified in duplex PCR reactions; recovery was normalized to an amplicon from the *ovalbumin (Ova)* gene, which is not expressed in B cells; and enrichment was normalized to a total DNA input control (see Materials and Methods for details). The distinct genomic structure of Vλ_R_ and Vλ_U_ permit them to be distinguished by PCR with specific primers. ChIP demonstrated considerable enrichment of acetylated histones H3 and H4 (AcH3 and AcH4) at the rearranged Vλ_R_ gene. In a typical experiment, AcH3 was enriched more than 80-fold at Vλ_R_, and AcH4 more than 30-fold (Figure 1B). In contrast, at the Vλ_U_ allele, the levels of AcH3 and AcH4 were much lower than at Vλ_R_ (16-fold and 7-fold lower, respectively), and only a few fold enriched relative to input DNA.

Chromatin structure within the ψVλ array was assayed by amplification with primers that interrogated seven sites, including a region between ψVλ1 and the Vλ gene, ψVλ1, ψVλ5, ψVλ13, ψVλ18, ψVλ24, ψVλ25, and the upstream flanking region. (Because of a paucity of polymorphisms, the ψVλ arrays at the two Igλ alleles in DT40 cannot be readily distinguished by PCR.) Strikingly, we observed considerable enrichment of AcH3 and AcH4 throughout the ψVλ array (Figure 1B). Enrichment was not proportional to distance from the transcribed Vλ_R_ gene, as sites distant from Vλ_R_ did not consistently display lower levels of enrichment than proximal sites (Figure 1B). Thus, enrichment of acetylated histones within the ψVλ array does not simply represent a graded spreading of chromatin modification from the transcribed Vλ_R_ gene to sites upstream. The nonuniform chromatin structure of the locus suggests the presence of *cis*-elements that regulate chromatin structure at the ψVλ array.

### Reversible Tethering of Lactose Repressor Fusion Proteins to the ψVλ Array in DT40 PolyLacO-λ_R_

**Figure 2:**
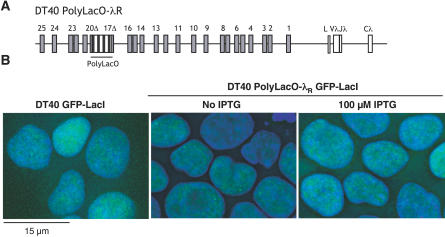
Reversible Tethering of GFP-LacI to the ψVλ Array in DT40 PolyLacO-λ_R_ (A) Schematic diagram of the rearranged chicken Igλ locus in DT40, with PolyLacO inserted between ψVλ17 and ψVλ20. Notations as in Figure 1. (B) Fluorescent images of DT40 GFP-LacI transfectants and DT40 PolyLacO-Vλ_R_ GFP-LacI transfectants cultured in the absence of IPTG (center) or in the presence of 100 μM IPTG overnight (right).

Local modification of chromatin structure can be achieved by tethering regulators to DNA binding sites as appropriate fusion proteins. This strategy has, for example, been used to show that the heterochromatin protein HP1, expressed as a fusion with Escherichia coli lactose repressor (LacI-HP1), promotes a closed chromatin structure and inactivation of reporter genes neighboring a LacO repeat in *Drosophila* [61,62], and to show that tethering of the vertebrate G9a histone methyltransferase to a GAL4 binding site within V(D)J minigene reporter impairs nonhomologous-mediated recombination of that construct [63]. Our laboratory has recently constructed a cell line, DT40 PolyLacO-λ_R_, that is a DT40 derivative in which PolyLacO has been inserted by homologous gene targeting between ψVλ17 and ψVλ20, 17 kb upstream of the transcribed Vλ_R_ (Figure 2A; M. Yabuki, E. C. Ordinario, W. J. Cummings, R. P. Larson, M. M. Fujii, et al., unpublished data). The PolyLacO insert is 3.8 kb in length and composed of approximately 100 copies of a 20-mer operator [64]. Using this cell line, it is possible to assay the effects of tethered regulatory factors on homologous recombination in a physiological process within an endogenous locus, avoiding the need for a transgene reporter. Control experiments have shown that the PolyLacO tag does not affect cell proliferation, cell cycle, or Ig gene diversification (M. Yabuki, E. C. Ordinario, W. J. Cummings, R. P. Larson, M. M. Fujii, et al., unpublished data).

In DT40 PolyLacO-λ_R_ GFP-LacI cells, which stably express enhanced green fluorescent protein (GFP) fused to LacI (GFP-LacI), the tagged λ_R_ allele can be directly imaged by fluorescence microscopy and appears as a distinct dot in each cell (Figure 2B, center). Tethering is reversible, as bright dots are not evident following overnight culture with 100 μM isopropyl-β-D-thiogalactoside (IPTG), which prevents LacI from binding to PolyLacO (Figure 2B, right).

### Tethered HP1 Diminishes Modifications Characteristic of Active Chromatin at ψVλ

**Figure 3:**
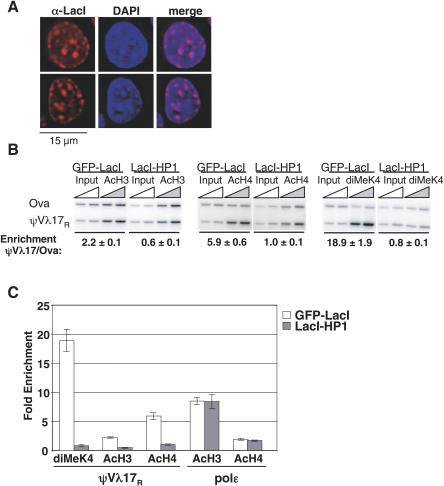
Tethered HP1 Diminishes Modifications Characteristic of Active Chromatin (A) Representative fluorescent images of single DT40 PolyLacO-Vλ_R_ LacI-HP1 transfectants, stained with anti-LacI antibodies (left), DAPI (center), or merged image (right). (B) Enrichment of AcH3 and AcH4 at ψVλ17_R_ in DT40 PolyLacO-Vλ_R_ GFP-LacI and DT40 PolyLacO-Vλ_R_ LacI-HP1 transfectants. Following ChIP, duplex PCR was carried out with *Ova* and ψVλ17_R_ primers. Enrichment is expressed relative to the total DNA input control ± standard deviation of four separate amplifications of increasing amounts of template DNA. (C) Histogram showing enrichment of AcH3 and AcH4 at ψVλ17_R_ (from [B]) and the *polɛ* promoter in DT40 PolyLacO-Vλ_R_ GFP-LacI and DT40 PolyLacO-Vλ_R_ LacI-HP1 transfectants. Bars indicate standard deviation.

To manipulate chromatin structure at the ψVλ array, we generated stable transfectants of DT40 PolyLacO-λ_R_ that express the D. melanogaster HP1 protein fused to LacI (LacI-HP1). HP1 is a nonhistone heterochromatin protein that functions in heterochromatic gene silencing, the spreading of heterochromatin, and histone deacetylation [58–60]. Tethered HP1 has been shown to promote a closed chromatin structure at adjacent genes [61,62,65–67]. Staining DT40 PolyLacO-λ_R_ LacI-HP1 transfectants with anti-LacI antibodies showed that LacI-HP1 colocalized with DAPI-dense regions corresponding to pericentric heterochromatin (Figure 3A), behaving as a functional marker of heterochromatin [68].

To ask if tethered LacI-HP1 altered chromatin structure, we assayed chromatin modifications at ψVλ17. This is the only site in the ψVλ array at which the rearranged and unrearranged alleles could be readily distinguished by use of specific PCR primers. Following ChIP, DNA was amplified with PCR primers specific for the targeted rearranged allele (ψVλ17_R_). Enrichment of ψVλ17_R_ was compared to the nonexpressed *Ova* gene as an internal control, and normalized to the ψVλ17_R_:*Ova* enrichment ratio in total input DNA (see Material and Methods). AcH3 and AcH4 were enriched 2.2-fold and 5.9-fold, respectively, at ψVλ17_R_ in DT40 PolyLacO-λ_R_ GFP-LacI controls (Figure 3B and 3C). These levels of enrichment are comparable to those documented in DT40 (Figure 1B). (Note that analysis of modification at ψVλ in the survey of the parental DT40 line necessarily included both alleles, which may underestimate activating modifications at the rearranged allele. In contrast, analysis of modifications at ψVλ17_R_ interrogates only the active allele.) AcH3 and AcH4 were not enriched at ψVλ17_R_ in DT40 PolyLacO-λ_R_ LacI-HP1 transfectants (0.6- and 1.0-fold, respectively; Figure 3B and 3C), consistent with HP1-mediated silencing. HP1 can effect silencing by recruitment of a histone methyltransferase that modifies lysine 9 of histone H3 [65–67], but may also promote silencing independently of this modification [61]. ChIP using antibodies against either di- and trimethylated H3 (lysine 9) did not reveal clear enrichment of the H3 lysine 9 methylation modification (data not shown). Dimethylation of lysine 4 of histone H3 (diMeK4[H3]) is associated with transcription and generally exhibits an overlapping distribution with acetylation [69,70]. Assays of diMeK4(H3) at ψVλ17_R_ demonstrated that this modification was 18.9-fold enriched in DT40 PolyLacO-λ_R_ GFP-LacI cells, but at background levels in DT40 PolyLacO-λ_R_ LacI-HP1 cells (Figure 3B and 3C).

HP1 promotes maintenance and spreading of heterochromatin [65]. To verify that changes in chromatin structure promoted by tethered HP1 did not spread throughout the chromosome, we examined another site near the Igλ locus on Chromosome 15, the gene encoding the catalytic subunit of DNA polɛ. DNA polɛ is ubiquitously expressed and essential for chromosomal replication in eukaryotes [71], and it is encoded by a gene mapping approximately 2.1 Mb from Igλ. We found no difference in enrichment of AcH3 at the *polɛ* promoter region in the DT40 PolyLacO-λ_R_ LacI-HP1 transfectants relative to DT40 PolyLacO-λ_R_ GFP-LacI controls (*polɛ/Ova* enrichment 8.5-fold and 8.4-fold, respectively; Figure 3C). Similarly, there was no difference in AcH4 at the *polɛ* promoter in the DT40 PolyLacO-λ_R_ LacI-HP1 transfectants relative to DT40 PolyLacO-λ_R_ GFP-LacI controls (*polɛ/Ova* enrichment 1.9-fold and 1.7-fold, respectively; Figure 3C). Thus, tethering of LacI-HP1 at ψVλ caused local modifications in chromatin structure, diminishing the AcH3, AcH4, and diMeK4(H3) modifications characteristic of open chromatin at ψVλ17_R_, and causing chromatin to adopt a less permissive state.

### Tethered HP1 Does Not Affect Vλ Gene Expression

**Figure 4:**
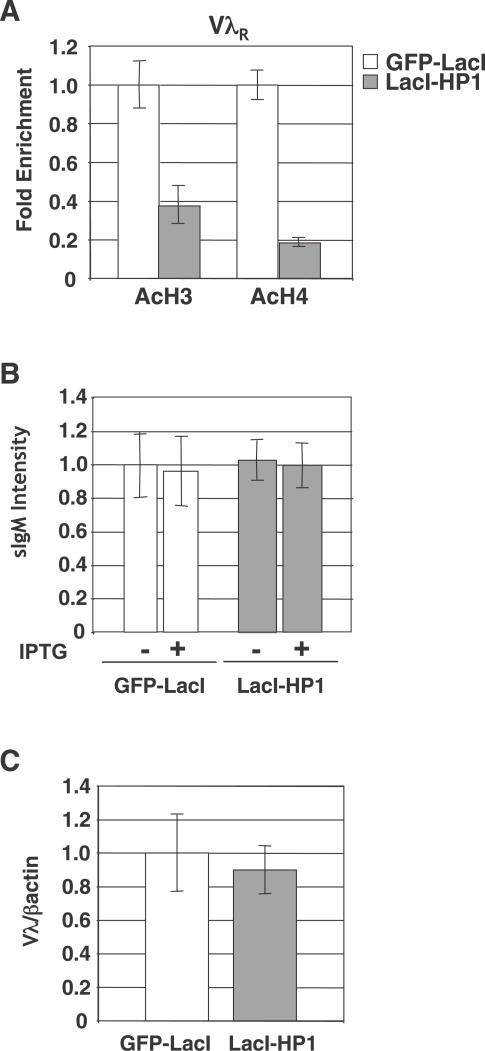
Tethered HP1 Does Not Affect Igλ Expression (A) Relative enrichment of AcH3 and AcH4 at Vλ_R_ in DT40 PolyLacO-Vλ_R_ GFP-LacI and DT40 PolyLacO-Vλ_R_ LacI-HP1 transfectants. Enrichment values were normalized to the DT40 PolyLacO-Vλ_R_ GFP-LacI control. Bars indicate standard deviation of four separate amplifications of increasing amounts of template DNA. (B) Relative intensity of sIgM expression in DT40 PolyLacO-λ_R_ GFP-LacI and DT40 PolyLacO-λ_R_ LacI-HP1 transfectants cultured in the presence or absence of 250 μM IPTG. sIgM levels were quantitated by measuring intensity of staining with mouse anti-chicken IgM antibody, and normalized to the level in DT40 PolyLacO-λ_R_ GFP-LacI transfectants. Details as in (A). (C) Relative levels of Vλ_R_ transcripts in DT40 PolyLacO-λ_R_ GFP-LacI and DT40 PolyLacO-λ_R_ LacI-HP1 transfectants. Transcript levels were quantitated by reverse transcriptase–PCR and normalized to the level in DT40 PolyLacO-λ_R_ GFP-LacI transfectants. Details as in (A).

We asked how tethered HP1 affected AcH3 and AcH4 levels at the expressed Vλ_R_ by comparing these modifications in DT40 PolyLacO-λ_R_ LacI-HP1 cells and the DT40 PolyLacO-λ_R_ GFP-LacI control transfectants (Figure 4A). Tethered HP1 diminished AcH3 and AcH4 levels to approximately 40% and 20% of the control levels, respectively. To ask if this affected gene expression, we assayed both surface IgM (sIgM) expression and Vλ transcript levels. Staining cells with mouse anti-chicken IgM showed that sIgM expression was comparable in DT40 PolyLacO-λ_R_ GFP-LacI and DT40 PolyLacO-λ_R_ LacI-HP1 lines, cultured in either the presence or absence of IPTG (Figure 4B). Vλ transcript levels were assayed in RNA harvested from DT40 PolyLacO-λ_R_ GFP-LacI and DT40 PolyLacO-λ_R_ LacI-HP1 cells, and normalized to *β-actin* as a control (Figure 4C). No significant difference was observed between Vλ transcript levels in the two cell lines, demonstrating that transcription is not affected by tethering of HP1 within the ψVλ array. Thus tethered LacI-HP1 did not affect expression of the downstream Ig gene, although it did diminish AcH3 and AcH4 levels at Vλ_R_. The very high AcH3 and AcH4 levels characteristic of Vλ (Figures 1B and 4A) are therefore not essential to maintain high levels of gene expression.

### Tethered HP1 Alters Local Chromatin Structure

**Figure 5:**
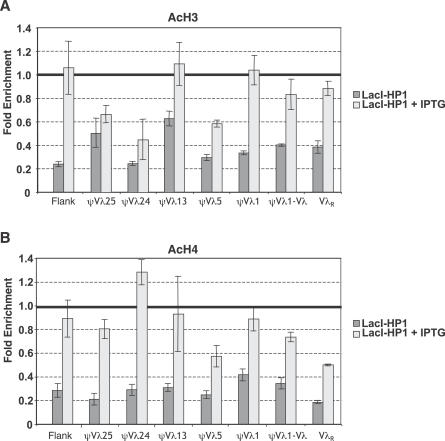
Tethered HP1 Decreases Histone Acetylation throughout the ψVλ Array (A) Summary of a ChIP experiment, assaying N-terminal acetylation of histone H3 in chromatin from the DT40 PolyLacO-Vλ_R_ LacI-HP1 cell line cultured for 3 d in the presence or absence of 250 μM IPTG. ChIP enrichment values (see Material and Methods) were normalized to values obtained from a parallel analysis of chromatin from DT40 PolyLacO-Vλ_R_ GFP-LacI cells. Bars indicate standard deviation of four separate amplifications of increasing amounts of template DNA. (B) Summary of a ChIP experiment, assaying N-terminal acetylation of histone H4 in chromatin from the DT40 PolyLacO-Vλ_R_ LacI-HP1 cell line cultured for 3 d in the presence or absence of 250 μM IPTG. Details as in (A).

To assess how extensive the chromatin effects of LacI-HP1 were, we examined AcH3 and AcH4 levels throughout the Igλ locus at the same amplicons examined in Figure 1, including one in the flank, six in the ψVλ array, and one at the expressed Vλ. Levels of modification were determined by comparing ψVλ17_R_:*Ova* ratios of immunoprecipitated and input conditions, as in Figure 3B. AcH3 modifications at the sites surveyed ranged from 24% to 63% of the levels at the same sites in the controls (Figure 5A, dark bars), and the average level of H3 acetylation across all of the sites was 38% of the DT40 PolyLacO-λ_R_ GFP-LacI control. Culture of DT40 PolyLacO-λ_R_ LacI-HP1 transfectants for 3 d with 250 μM IPTG increased acetylation of H3 at all eight sites surveyed (Figure 5A, compare dark and light bars). The effects of IPTG culture were somewhat variable, but at most sites IPTG culture restored levels of AcH3 to at least 45% of the level in the DT40 PolyLacO-λ_R_ GFP-LacI control cells, with an average of over 80%. Thus, the chromatin modifications at ψVλ17_R_ in DT40 PolyLacO-λ_R_ LacI-HP1 cells resulted directly from tethered LacI-HP1, and were largely reversible.

H4 acetylation was surveyed at the same eight sites (Figure 5B, dark bars). AcH4 modifications were found to range from 18% to 42% of control levels, and the average level was 29% of that of the control cell line. Culture with IPTG for 3 d increased acetylation of H4 at all eight sites surveyed (Figure 5B, compare dark and light bars), restoring H4 acetylation to at least 57% of the level in the DT40 PolyLacO-λ_R_ GFP-LacI control cells, with an average of over 80%. Moreover, IPTG can at least partially reverse the effects of LacI-HP1.

These results show that the observed chromatin modifications in the ψVλ array are due to tethering of HP1. Moreover, the fact that these modifications are reversible shows that an active mechanism reverses histone modifications imposed by tethering chromatin modification factors at ψVλ.

### Tethered HP1 Impairs Templated Mutagenesis

**Figure 6:**
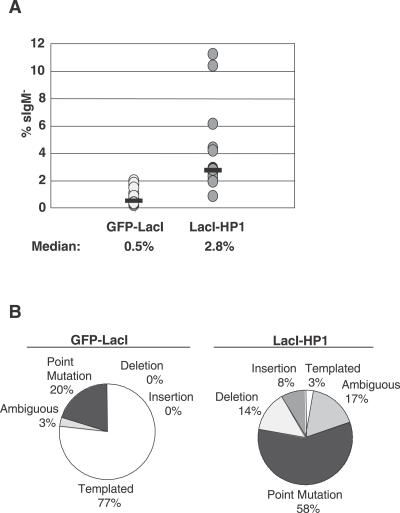
Nontemplated and Templated Mutation Promoted by Tethered HP1 (A) sIgM loss fluctuation assay of panels of independent DT40 PolyLacO-Vλ_R_ GFP-LacI (*n* = 27) and DT40 PolyLacO-Vλ_R_ LacI-HP1 (*n* = 16). The figure shows combined data from at least two independent transfectants for each fusion construct. Median diversification rates are shown below. (B) Summary of sequence analysis of Vλ regions carrying unique mutations from DT40 PolyLacO-Vλ_R_ GFP-LacI (*n* = 71) and DT40 PolyLacO-Vλ_R_ LacI-HP1 (*n* = 36) transfectants, as analyzed by single-cell PCR. Sequences were pooled from two independent transfectants.

The ability to manipulate chromatin structure at ψVλ by tethering LacI-HP1 (Figures 3–5) enabled us to directly ask whether and how chromatin structure influences Ig gene conversion. We used the sIgM loss variant assay to determine if tethered LacI-HP1 affected the clonal rate of sequence diversification of the rearranged Vλ_R_ gene. This fluctuation assay measures the fraction of variant cells that no longer express structurally intact sIgM, and thus scores mutation events resulting from either gene conversion or point mutagenesis [47,50]. Independent clonal derivatives of DT40 PolyLacO-λ_R_ GFP-LacI and DT40 PolyLacO-λ_R_ LacI-HP1 were established by limiting dilution cloning of sIgM^+^ cells; the fraction of sIgM^−^ cells in each population was determined by flow cytometry of cells cultured for 4 wk and then stained with anti-IgM antibody. The median sIgM loss rate was 0.5% for DT40 PolyLacO-λ_R_ GFP-LacI cells and 2.8% for DT40 PolyLacO-λ_R_ LacI-HP1 cells (Figure 6A). This corresponds to a 5.6-fold acceleration of clonal diversification rates in LacI-HP1 transfectants relative to GFP-LacI controls.

Ig gene diversification in chicken B cells occurs predominantly by gene conversion (templated mutation), but if gene conversion is impaired, for example by the absence of essential factors, repair can create a significant fraction of nontemplated mutations [50–55]. This is typically accompanied by an increase in the clonal diversification rate, because the ψVλ templates for gene conversion are about 80% identical to the rearranged gene, and a significant fraction of DNA lesions that are repaired by gene conversion do not undergo any alteration of sequence; in contrast, repair by a mutagenic polymerase is more likely to alter DNA sequence. To determine how tethering of HP1 accelerated diversification, we sorted single sIgM^−^ cells from the DT40 PolyLacO-λ_R_ GFP-LacI and DT40 PolyLacO-λ_R_ LacI-HP1 transfectants, amplified expressed Vλ regions by single-cell PCR, and sequenced these regions. Sequence changes were categorized as templated if they were within a tract containing two or more base changes and the tract was an exact match to at least 9 bp of a donor ψVλ sequence, and as ambiguous if they consisted of only a single base change while matching at least 9 bp of a donor ψVλ sequence. Nontemplated events, consisting of point mutations, deletions, and insertions, were also scored. In the control DT40 PolyLacO-λ_R_ GFP-LacI transfectants, 55 templated events and two ambiguous events were documented among 71 mutations; thus, most events (77%) were templated, and a small fraction of events (20%) were point mutations (Figure 6B, left; Figure S1A). Strikingly, in DT40 PolyLacO-λ_R_ LacI-HP1 cells, point mutations predominated (58%), accompanied by deletions (8%) and insertions (14%), while only one clearly templated event and six ambiguous events were documented among 36 mutations (Figure 6B, right; Figure S1B). Thus, only 3% of mutations were clearly templated, and even including the ambiguous class of potentially templated mutations, templating could account for no more than 19% of mutation. Statistical comparisons showed that the difference between the fraction of clearly templated mutations in DT40 PolyLacO-λ_R_ GFP-LacI control cells and DT40 PolyLacO-λ_R_ LacI-HP1 transfectants (77% compared to 3%) was highly significant (*p* = 7.5 × 10^−7^, Fisher's exact test). The difference in the fraction of ambiguous, potentially templated mutations in the control cells (3%) and HP1 transfectants (17%) is also significant (*p* = 0.05, Fisher's exact test). This suggests that some mutations in this category may arise as a result of limitations on the length of a gene conversion tract imposed by nonpermissive donor chromatin. Thus, tethering of HP1 accelerated clonal rates of mutagenesis by impairing templated mutation.

## Discussion

Gene conversion at the chicken Ig loci uses an array of upstream ψV donors as templates for homology-directed repair of lesions targeted to the rearranged and transcribed V genes. We have shown that in chicken B cells carrying out active Ig gene conversion, chromatin within the donor ψVλ array is characterized by enrichment of AcH3 and AcH4, modifications that correlate with an open chromatin structure. We directly demonstrated the importance of permissive chromatin structure for Ig gene conversion by showing that tethering the heterochromatin protein HP1 to the ψVλ donor array caused local changes in chromatin structure, diminishing the AcH3, AcH4, and diMeK4(H3) modifications characteristic of open chromatin. Although these changes were not accompanied by the lysine 9 methylation (H3) modification characteristic of closed chromatin, they caused the region to adopt a state less permissive for gene conversion. Tethering of HP1 was accompanied by a dramatic shift in the Ig Vλ mutation spectrum, so that templated mutations were in the minority and point mutations predominated. Importantly, this effect on mutagenesis was correlated with a change in chromatin structure and not changes in expression of the locus. Thus, chromatin structure can dictate whether gene conversion occurs at an endogenously generated DNA lesion.

### The Mechanism of Gene Conversion within a Complex Chromatin Landscape

Gene conversion at Vλ results from priming of new DNA synthesis at the 3′ end of a break using a ψVλ region as template. Gene conversion requires synapsis between the donor and recipient DNA, as well as access to the donor by factors that carry out homology-directed repair. The elevated levels of H3 and H4 acetylation characteristic of the ψVλ array in wild-type DT40 are evidence of a relaxed chromatin structure, which would increase the accessibility of the ψVλ genes to *trans*-acting factors and also create a three-dimensional architecture that is favorable for sequence synapsis.

HP1 tethered within the ψVλ donor array impaired gene conversion at the rearranged Vλ_R_, without affecting Vλ gene expression. Chromatin changes caused by tethered HP1 may impair gene conversion by impeding access of repair factors and the invading strand to the donor template. Tethered HP1 may also contribute to larger chromosomal architecture that affects the mechanics of DNA repair pathways, such as looping necessary to juxtapose donor and recipient sequences. The point mutations that accumulated in LacI-HP1 transfectants are typical of thwarted recombinational repair, and are characteristic of cells lacking either *trans*-acting factors essential for recombination [49–55] or some or all of the ψV donor array [56]. HP1 regulates chromatin structure and heterochromatic gene silencing in two ways, by partnering with a histone methyltransferase [65] and by recruiting histone deacetylases [60]. Tethered HP1 caused modification changes characteristic of a nonpermissive chromatin structure within ψVλ.

Histone acetylation has been documented at actively transcribed mammalian Ig genes undergoing somatic hypermutation and class switch recombination, but whether hyperacetylation contributes to targeting of diversification has yet to be resolved [72–76]. A connection between histone acetylation and gene conversion was suggested by experiments showing that treatment of DT40 cells with the histone deacetylase inhibitor trichostatin A promotes genome-wide histone deacetylation accompanied by increased gene conversion at Vλ_R_ [77]. However, the interpretation of those results is complicated by the fact that the effects of trichostatin A are genome-wide, and not specific. The DT40 PolyLacO-λ_R_ cell line permits local manipulation of chromatin structure, avoiding that complication. Moreover, we were able to demonstrate that the effects of tethering a LacI-HP1 fusion protein were largely reversed upon culture with IPTG, so an active mechanism must determine chromatin modification at ψVλ. For studies of homologous recombination, the DT40 PolyLacO-λ_R_ B cell line has the further advantage that Ig gene conversion is a physiological process within an endogenous locus, avoiding the need for a transgene reporter.

### Chromatin Structure, Genome Stability, Aging, and Gene Therapy

The importance of chromatin structure to the outcome of homologous recombination has implications for understanding the mechanisms that normally maintain genomic stability. There are vast numbers of repetitive elements distributed throughout the vertebrate genome, and recombination between these elements can lead to genomic instability [78]. In the human genome, there are approximately one million Alu elements, and recombination between Alu elements can cause duplications leading to tumorigenesis and genetic disease [79,80]. Histones carrying repressive modifications are enriched at repetitive elements [81]. These modifications undoubtedly maintain transcriptional repression; our results suggest they may also contribute to suppression of recombination.

LOH occurs as a result of unequal mitotic recombination between homologs at allelic sites. The mechanism of LOH is of particular interest, because it contributes to loss of tumor suppressor gene function, leading to tumorigenesis [82]. Recent experiments have demonstrated an age-dependent increase in LOH in Saccharomyces cerevisiae [83] and in reporter genes in *Drosophila* germ cells [84], and an increase in homologous recombination in mouse pancreatic cells [85]. Mechanisms proposed to explain age-associated LOH include elevated rates of DNA damage, changes in the cell cycle distribution, and inactivation of homology-independent repair pathways with aging. Our results suggest another possibility, that relaxation of chromatin structure may accompany aging and promote a genome-wide increase in homologous recombination in aging cells. This possibility is supported by recent analysis of *Drosophila* [86], as well as by recent evidence that the mutant form lamin A polypeptide (product of the human *LMNA* gene) responsible for Hutchinson-Gilford progeria syndrome leads to a genome-wide loss of H3 methylation [87].

The finding that chromatin structure regulates homologous recombination also has practical ramifications. Considerable current effort is directed toward developing strategies that harness a cell's capacity for homology-dependent repair to promote gene therapy, by providing an intact donor gene to replace a deficient target gene [88]. Our results suggest that permissive structure at the donor will be an important design parameter in developing donor genes for therapeutic applications.

## Materials and Methods

### Chromatin immunoprecipitation.

ChIP was carried out as previously described [48,89]. For all experiments at least two chromatin preparations from at least two independent stably transfected lines were analyzed. Figures present one representative experiment in which results from analysis of four separate amplifications were used to calculate a standard deviation. Enrichment of the experimental amplicon was normalized to enrichment of an internal control amplicon from the *Ova* gene, amplified in the same tube by duplex PCR, and enrichment upon ChIP with specific antibodies was normalized to parallel experiments in which ChIP was carried out with total input DNA controls. Inclusion of the *Ova* internal control amplicon enabled us to normalize for immunoprecipitation efficiency, background carryover, and differences in gel loading. Enrichment equaled [(ψVλ/*Ova*)_Ab_]/[(ψVλ/*Ova*)_Input_]. As an additional control, the ratio of the experimental and control amplicons in the total input control was compared to a control ChIP with polyspecific IgG; in all cases, enrichment in input and IgG controls were essentially equal.

Antibodies used were as follows: anti-AcH3 (06–599), anti-AcH4 (06–866), and diMeK4(H3) (07–030) from Upstate ( http://www.upstate.com/). PCR primers for ChIP were as follows: Vλ_R_, 5′-GCCGTCACTGATTGCCGTTTTCTCCCCTC-3′ and 5′-CGAGACGAGGTCAGCGACTCACCTAGGAC-3′; region between ψVλ1 and Vλ, 5′-CTGTGGCCTGTCAGTGCTTA-3′ and 5′-GCAGGGAACCACAAGAACAT-3′; ψVλ1, 5′-GGGACTTGTGTCACCAGGAT-3′ and 5′-CGCAGTCACATGTGGAATATC-3′; ψVλ5, 5′-GAGCCCCATTTTCTCTCCTC-3′ and 5′-GAGATGTGCAGCAACAAGGA-3′; ψVλ13, 5′-CCCTCTCCCTATGCAGGTTC-3′ and 5′-CCCCTATCACCATACCAGGA-3′; ψVλ18, 5′-CCATTTTCTCCCCTCTCTCC-3′ and 5′-TCACCCTACAGCTTCAGTGC-3′; ψVλ24, 5′-CCATTTTCTCCCCTCTCTCC-3′ and 5′-CAGCCCATCACTCCCTCTTA-3′; ψVλ25, 5′-TCTGTTGGTTTCAGCACAGC-3′ and 5′-GCAGTTCTGTGGGATGAGGT-3′; ψVλ upstream flank, 5′-GGCTCCTGTAGCTGATCCTG-3′ and 5′-GTTCTTTGCTCTTCGGTTGC-3′; ψVλ17 at the PolyLacO-targeted allele, 5′-TAGATAGGGATAACAGGGTAATAGC-3′ and 5′-AGGGCTGTACCTCAGTTTCAC-3′; *Ova,* 5′-ATTGCGCATTGTTATCCACA-3′ and 5′-TAAGCCCTGCCAGTTCTCAT-3′; and *polɛ,* 5′-GGGCTGGCTCATCAACAT-3′ and 5′-CTGGGTGGCCACATAGAAGT-3′.

### Constructs, transfection, and cell culture.

The LacI-HP1 expression plasmid was created by substituting LacI-HP1 from a construct provided by L. Wallrath (University of Iowa) for AID in pAIDPuro (from H. Arakawa, Munich, Germany), to position LacI-HP1 downstream of the chicken *β-actin* promoter. The GFP-LacI expression plasmid (p3′ss-EGFP-LacI) was provided by A. Belmont (University of Illinois). Cell culture and transfection were carried out as previously described [47]. DT40 PolyLacO-λ_R_ was generated by homologous gene targeting, using a construct carrying approximately 3.8 kb of PolyLacO flanked by arms designed for targeting the region between ψVλ17 and ψVλ20, 17 kb upstream of the transcribed Vλ_R_ (M. Yabuki, E. C. Ordinario, W. J. Cummings, R. P. Larson, M. M. Fujii, et al., unpublished data). In brief, homologous integrants were identified by PCR, and the selectable marker deleted by Cre expression. The DT40 bursal lymphoma derives from B cells in which only one Igλ allele is rearranged, and in which the two parental chromosomes are distinguished by a polymorphism near ψVλ17. This enabled us to determine whether the rearranged or unrearranged allele had been targeted by PCR. Control experiments established that cell cycle distribution was comparable in DT40 PolyLacO-λ_R_, DT40 PolyLacO-λ_R_ GFP-LacI, and DT40 PolyLacO-λ_R_ LacI-HP1 cells, and that culture of cells with up to 500 μM IPTG for 7 d did not affect proliferation rate or chromatin modifications at ψVλ17_R_ in DT40 PolyLacO-λ_R_ GFP-LacI control cells. Oligonucleotides for Vλ sequence analysis have been described [47].

### Fluorescence imaging.

For fluorescence imaging, cells (2 × 10^5^) were cytospun onto glass slides and fixed with 2% paraformaldehyde for 20 min, permeabilized with 0.1% NP-40 for 15 min, and stained as previously described [90]. Primary staining was with an anti-LacI monoclonal antibody (1:500 dilution; Upstate), and the secondary antibody was donkey anti-mouse IgG Alexa Fluor 594 (1:2,000; Molecular Probes, http://probes.invitrogen.com/). To visualize the nucleus, cells were stained with DAPI (Sigma-Aldrich, http://www.sigmaaldrich.com/). Fluorescent images were acquired using the DeltaVision microscopy system (Applied Precision, http://www.appliedprecision.com/) and processed with softWoRx software (Applied Precision).

### Reverse transcriptase–PCR.

RNA was harvested from cells using TRIzol Reagent (Invitrogen, http://www.invitrogen.com/), purified with a PreAnalytiX column (Qiagen, http://www1.qiagen.com/), and subject to one round of reverse transcription prior to PCR. Vλ transcripts were PCR-amplified following dilution of the template (1:1,300), and *β-actin* was PCR-amplified from an undiluted sample. The primers for amplification of Vλ were 5′-GTCAGCAAACCCAGGAGAAAC-3′ and 5′-AATCCACAGTCACTGGGCTG-3′. The primers for amplification of *β-actin* have been described [36].

### Quantitation of sIgM loss variants and sequence analysis.

The sIgM loss variant assay, which measures the accumulated sIgM loss variants resulting from frameshift or nonsense mutations in mutated V regions, was used to quantitate Ig V region diversification [47,50]. In brief, sIgM^+^ cells were isolated by flow cytometry followed by limiting dilution cloning, and expanded for 4 wk. To quantitate the fraction of sIgM^−^ cells, approximately 1 × 10^6^ cells were stained with anti-chicken IgM-RPE (SouthernBiotech, http://www.southernbiotech.com/) and analyzed on a FACScan with CellQuest software (BD Biosciences, http://www.bdbiosciences.com/).

Single-cell PCR and sequence analysis were performed as described [47]. In brief, sIgM^−^ cells were sorted and aliquoted to single wells, Vλ regions were amplified and sequenced, and their sequences were compared to those of the ψVλ donors to determine if mutations were templated or nontemplated. The criterion for a templated mutation was that nine consecutive bases must be an exact match in donor and recipient. Sequences were derived from two independently transfected lines. Only unique sequences were included for classification of the mutations.

## Supporting Information

Figure S1 Sequence Alignment of Mutated DT40 PolyLacO-λ_R_ GFP-LacI and DT40 PolyLacO-λ_R_ LacI-HP1 Clones Sequences of unique, mutated Vλ regions from diversified (A) DT40 PolyLacO-λ_R_ GFP-LacI and (B) DT40 PolyLacO-λ_R_ LacI-HP1 cells. Clear blue boxes outline gene conversion tracts; red circles denote point mutations; black dotted boxes indicate nontemplated insertions; orange triangles denote deletions; blue-shaded boxes indicate ambiguous changes.
(83 KB PDF)

## Abbreviations

ψV: pseudo-variable
AcH3: acetylated histone H3
AcH4: acetylated histone H4
ChIP: chromatin immunoprecipitation
diMeK4(H3): histone H3 dimethylated at lysine 4
DSB: double-strand break
GFP: green fluorescent protein
Ig: immunoglobulin
Igλ: immunoglobulin light chain
IPTG: isopropyl-β-D-thiogalactoside
LOH: loss of heterozygosity
PolyLacO: polymerized lactose operator
sIgM: surface immunoglobulin M
V: variable
