# Supplementary material for: Chromatin Structure Regulates Gene Conversion
Source: PLoS Biol. 2007 Sep 18;5(10):e246. doi: 10.1371/journal.pbio.0050246 (PMC1976632; doi:10.1371/journal.pbio.0050246)
Supplement: Supplementary file 1 [file pbio.0050246.sg001.pdf]

LacI-GFP

```
DT40     TCT CCC TCT CCA GGT TCC CTG GTG CAG GCA GCG CTG ACT CAG CCG GCC TCG GTG TCA GCA AAC CTG
GFP-1    --- --- --- --- --- --- --- --- --- --- --- --- --- --- --- --- --- --- --- --- --- ---
GFP-2    --- --- --- --- --- --- --- --- --- --- --- --- --- --- --- --- --- --- --- --- --- -CA
GFP-3    --- --- --- --- --- --- --- --- --- --- --- --- --- --- --- --- --- --- --- --- --- -CA
GFP-4    --- --- --- --- --- --- --- --- --- --- --- --- --- --- --- --- --- --- --- --- --- -CA
GFP-5    --- --- --- --- --- --- --- --- --- --- --- --- --- --- --- --- --- --- --- --- --- -CA
GFP-6    --- --- --- --- --- --- --- --- --- --- --- --- --- --- --- --- --- --- --- --- --- -CA
GFP-7    --- --- --- --- --- --- --- --- --- --- --- --- --- --- --- --- --- --- --- --- --- -CA
GFP-8    --- --- --- --- --- --- --- --- --- --- --- --- --- --- --- --- --- --- --- --- --- -CA
GFP-9    --- --- --- --- --- --- --- --- --- --- --- --- --- --- --- --- --- --- --- --- --- -CA
GFP-10   --- --- --- --- --- --- --- --- --- --- --- --- --- --- --- --- --- --- --- --- --- -CA
GFP-11   --- --- --- --- --- --- --- --- --- --- --- --- --- --- --- --- --- --- --- --- --- -CA
GFP-12   --- --- --- --- --- --- --- --- --- --- --- --- --- --- --- --- --- --- --- --- --- -CA
GFP-13   --- --- --- --- --- --- --- --- --- --- --- --- --- --- --- --- --- --- --- --- --- -CA
GFP-14   --- --- --- --- --- --- --- --- --- --- --- --- --- --- --- --- --- --- --- --- --- -CA
GFP-15   --- --- --- --- -G  AA- --- --- --- --- --- --- --- --- --- --- --- --- --- --- --- -CA
GFP-16   --- --- --- --- --- --- --- --- --- --- --- --- --- --- --- --- --- --- --- --- --- -CA
GFP-17   --- --- --- --- --- --- --- --- --- --- --- --- --- --- --- --- --- --- --- --- --- -CA
GFP-18   --- --- --- --- --- --- --- --- --- --- --- --- --- --- --- --- --- --- --- --- --- -CA
GFP-19   --- --- --- --- --- --- --- --- --- --- --- --- --- --- --- --- --- --- --- --- --- -CA

DT40     GGA GGA ACC GTC AAG ATC ACC TGC TCC GGG GGT GGC AGC TAT GCT GGA AGT TAC TAT TAT GGC TGG
GFP-1    --- --- --- --- --- --- --- --- --- --- --- --- --- --- --- --- -C- --- --- --- --- ---
GFP-2    --- -A- --- --- --- --- --- --- --- --- --- --- -C- --- --- --- --- --- --- --- --- ---
GFP-3    --- -A- --- --- --- --- --- --- --- --- --- --- --- --- --- --- --- --- --- --- --- ---
GFP-4    --- -A- --- --- --- --- --- --- --- --- --- --- --- --- --- --- --- --- --- --- --- ---
GFP-5    --- -A- --- --- --- --- --- --- --- --- --- --- --- --- --- --- --- --- --- --- --- ---
GFP-6    --- -A- --- --- --- --- --- --- --- --- --- --- --- --- --- --- --- --- --- --- --- ---
GFP-7    --- -A- --- --- --- --- --- --- --- --- --- --- --- --- --- --- --- --- --- --- --- ---
GFP-8    --- -A- --- --- --- --- --- --- --- --- --- --- --- --- --- --- --- --- --- --- --- ---
GFP-9    --- -A- --- --- --- --- --- --- --- --- --- --- -G- --- --- --- --- --- --- --- --- ---
GFP-10   --- -A- --- --- --- --- --- --- --- --- --- --- --- --- --- --- --- --- --- --- --- ---
GFP-11   --- -A- --- --- --- --- --- --- --- --- --- --- --- --- --- --- --- --- --- --- --- ---
GFP-12   --- -A- --- --- --- --- --- --- --- --- --- --- --- --- --- --- --- --- --- --- --- ---
GFP-13   --- -A- --- --- --- --- --- --- --- --- --- --- --- --- --- --- --- --- --- --- --- ---
GFP-14   --- -A- --- --- --- --- --- --- --- --- --- --- --- --- --- --- --- --- --- --- --- ---
GFP-15   --- -A- --- --- --- --- --- --- --- --- --- --- --- --- --- --- --- --- --- --- --- ---
                                                           GGC
                                                            ▼
GFP-16   --- -A- --- --- --- --- --- --- --- --- --- --- --- --- --- --- --- --- --- --- --- ---
                                                            AGC
                                                            ▼
GFP-17   --- -A- --- --- --- --- --- --- --- --- A-- -A- --- --- --- --- --- --- --- --- --- ---
GFP-18   --- -A- --- --- --- --- --- --- --- --- --- --- --- --C TA- --- --- --- --- --- --- -A
GFP-19   --- -A- --- --- --- --- --- --- --- --- --- --- --- --- --- --- --- --- --- --- --- ---

DT40     TAC CAG CAG AAG TCT CCT GGC AGT GCC CCT GTC ACT GTG ATC TAT GAC AAC GAC AAG AGA CCC TCG
GFP-1    --- --- --- --- --- --- --- --- --- --- --- --- --- --- --- --- --- --- --- --- --- ---
GFP-2    --- --- --- --- --- --- --- --- --- --- --- --- --- --- --- --- --- --- --- --- --- ---
GFP-3    --- --- --- --- G-A --- --- --- --- --- --- --- C-- --- --- T-- --- A-- --- --- --- ---
GFP-4    --- --- --- --- G-A --- --- --- --- --- --- --- C-- --- --- T-- --- A-- --- --- --- ---
GFP-5    --- --- --- --- G-A --- --- --- --- --- --- --- C-- --- --- T-- --- A-- --- --- --- ---
GFP-6    --- --- --- --- -A- --- --- --- --- --- --- --- C-- --- --- T-- --- A-- --- --- --- ---
GFP-7    --- --- --- --- G-A --- --- --- --- --- --- -G- C-- --- --- T-- --- A-- --- --- --- ---
GFP-8    --- --- --- --- G-A --- --- --- --- --- --- --- C-- --- --- T-- --- A-- --- --- --- ---
GFP-9    --- --- --- --- G-A --- --- --- --- --- --- --- C-- --- --- T-- --- A-- --- --- --- ---
GFP-10   --- --- --- --- G-A --- --- --- --- --- --- --- C-- --- --- T-- --- A-- --- --- --- ---
GFP-11   --- --- --- --- G-A --- --- --- --- --- --- --- --- --- --- AG- --- A-- --- --- --- ---
GFP-12   --- --- --- --- G-A --- --- --- --- --- --- --- --- --- --- T-- --- A-- --- --- --- ---
GFP-13   --- --- --- --- G-A --- A-- --- -T- --- --- --- C-- --- --- T-- --- A-- --- --- --- ---
GFP-14   --- --- --- --- G-A --- --- --- --- --- --- --- C-- --- --- T-- --- A-- --- --C --- ---
GFP-15   --- --- --- --- G-A --- --- --- --- --- --- --- C-- --- --- T-- --- A-- --- --- --- ---
GFP-16   --- --- --- --- G-A --- --- --- --- --- --- --- C-- --- --- T-- --- A-- --- --- --- ---
GFP-17   --- --- --- --- G-A --- --- --- --- --- --- --- C-- --- --- T-- --- A-- --- --- --- ---
GFP-18   --- --- --- --- G-A --- --- --- --- --- --- --- C-- --- --- T-- --- A-- --- --- --- ---
GFP-19   --- --- --- --- G-A --- --- --- --- --- --- --- --- --- --- --- --- AC- --- --C --- ---
```

```
DT40    GAC ATC CCT TCA CGA TTC TCC GGT TCC CTA TCC GGC TCC ACA AAC ACA TTA ACC ATC ACT GGG GTC
GFP-1   --- --- --- --- --- --- --- --- --- --- --- --- --- --- --- --- --- --- --- --- --- ---
GFP-2   --- --- --- --- --- --- --- --- --- --- --- --- --- --- --- --- --- --- --- --- --- ---
GFP-3   --- --- --- --- --- --- A-- --- --- --- --- --- --- --- --- --- --- --- --- --- --- ---
GFP-4   --- --- --- --- --- --- --- --- --- --- --- -A- --- --- --- --- --- --- --- --- --- ---
GFP-5   --- --- --- --- --- --- --- --- --- --- --- --- --- --- --- --- --- --- --- --- --- ---
GFP-6   --- --- --- --- --- --- --- --- --- --- --- --- --- --- --- --- --- --- --- --- --- ---
GFP-7   --- --- --- --- --- --- --- --- --- --- --- --- --- --- --- --- --- --- --- --- --- ---
GFP-8   --- --- --- --- --- --- --- --- --- --- --- --- --- --- --- --- --- --- --- --- --- ---
GFP-9   --- --- --- --- --- --- --- --- --- --- --- --- --- --- --- --- --- --- --- --- --- ---
GFP-10  --- --- --- --- --- --- --- --- --- --- --- --- --- --- --- --- --- --- --- --- --- ---
GFP-11  --- --- --- --- --- --- --- --- --- --- --- --- --- --- --- --- --- --- --- --- --- ---
GFP-12  --- --- --- --- --- --- --- --- --- --- --- --- --- --- --- --- --- --- --- --- --- ---
GFP-13  --- --- --- --- --- --- --- --- --- --- --- --- --- --- --- --- --- --- --- --- --- ---
GFP-14  --- --- --- --- --- --- --- --- --- --- --- --- --- --- --- --- --- --- --- --- --- ---
GFP-15  --- --- --- --- --- --- --- --- --- --- --- --- --- --- --- --- --- --- --- --- --- ---
GFP-16  --- --- --- --- --- --- --- --- --- --- --- --- --- --- --- --- --- --- --- --- --- ---
GFP-17  --- --- --- --- --- --- --- --- --- --- --- --- --- --- --- --- --- --- --- --- --- ---
GFP-18  --- --- --- --- --- --- --- --- --- --- --- --- --- --- --- --- --- --- --- --- --- ---
GFP-19  A-- --- --- --- --- --- --- --- --- --- --- --- --- --- --- --- --- --- --- --- --- ---

DT40    CGA GCC GAT GAC GAG GCT GTC TAT TTC TGT GGG AGT GCA GAC AAC AGT GGT GCT GCA TTT GGG GCC
GFP-1   --- --- --- --- --- --- --- --- --- --- --- --- --- --- --- --- --- --- --- --- --- ---
GFP-2   --- --- --- --- --- --- --- --- --- --- --- --- --- --- --- --- --- --- --- --- --- ---
GFP-3   --- --- --- --- --- --- --- --- --- --- --- --- --- --- --- --- --- --- --- --- --- ---
GFP-4   --- --- --- --- --- --- --- --- --- --- --- --- --- --- --- --- --- --- --- --- --- ---
GFP-5   --- --- --- --- --- --- --- --- --- --- --- --- T-- --- --- --- --- --- --- --- --- ---
GFP-6   --- --- --- --- --- --- --- --- --- --- --- --- --- --- --- --- --- --- --- --- --- ---
GFP-7   --- --- --- --- --- --- --- --- --- --- --- --- --- --- --- --- --- --- --- --- --- ---
GFP-8   --- --- --- --- --- --- --- --- --- --- --- --- --- --- --- --- --- A-- A-- --- --- ---
GFP-9   --- --- --- --- --- --- --- --- --- --- --- --- --- --- --- --- --- --- --- --- --- ---
GFP-10  --- --- --- --- --- --- --- --- -A- --- --- --- --- --- --- -C- --- --- --- --- --- ---
GFP-11  --- --- --- --- --- --- --- --- --- --- --- --- --- --- --- --- --- --- --- --- --- ---
GFP-12  --- --- --- --- --- --- --- --- --- --- --- --- --- --- --- --- --- --- --- --- --- ---
GFP-13  --- --- --- --- --- --- --- --- --- --- --- --- --- --- --- --- --- --- --- --- --- ---
GFP-14  --- --- --- --- --- --- --- --- --- --- --- --- --- --- --- --- --- --- --- --- --- ---
GFP-15  --- --- --- --- --- --- --- --- --- --- --- -C- --- --- --- --- --- --- --- --- --- ---
GFP-16  --- --- --- --- --- --- --- --- --- --- --- --- --- --- --- --- --- --- --- --- --- ---
GFP-17  --- --- --- --- --- --- --- --- --- --- --- --- --- --- --- --- --- --- --- --- --- ---
GFP-18  --- --- --- --- --- --- --- --- --- --- --- --- --- --- --- --- --- --- --- --- --- ---
GFP-19  --- --- --- --- --- --- --- --- --- --- --- --- --- --- --- --- --- --- --- --- --- ---

DT40    GGG ACA ACC CTG ACC GTC CTA GGT GAG TCG CTG ACC TCG TCT CGG TCT
GFP-1   --- --- --- --- --- --- --- --- --- --- --- --- --- --- --- ---
GFP-2   --- --- --- --- --- --- --- --- --- --- --- --- --- --- --- ---
GFP-3   --- --- --- --- --- --- --- --- --- --- --- --- --- --- --- ---
GFP-4   --- --- --- --- --- --- --- --- --- --- --- --- --- --- --- ---
GFP-5   --- --- --- --- --- --- --- --- --- --- --- --- --- --- --- ---
GFP-6   --- --- --- --- --- --- --- --- --- --- --- --- --- --- --- ---
GFP-7   --- --- --- --- --- --- --- --- --- --- --- --- --- --- --- ---
GFP-8   --- --- --- --- --- --- --- --- --- --- --- --- --- --- --- ---
GFP-9   --- --- --- --- --- --- --- --- --- --- --- --- --- --- --- ---
GFP-10  --- --- --- --- --- --- --- --- --- --- --- --- --- --- --- ---
GFP-11  --- --- --- --- --- --- --- --- --- --- --- --- --- --- --- ---
GFP-12  --- --- --- --- --- --- --- --- --- --- --- --- --- --- --- ---
GFP-13  --- --- --- --- --- --- --- --- --- --- --- --- --- --- --- ---
GFP-14  --- --- --- --- --- --- --- --- --- --- --- --- --- --- --- ---
GFP-15  --- --- --- --- --- --- --- --- --- --- --- --- --- --- --- ---
GFP-16  --- --- --- --- --- --- --- --- --- --- --- --- --- --- --- ---
GFP-17  --- --- --- --- --- --- --- --- --- --- --- --- --- --- --- ---
GFP-18  --- --- --- --- --- --- --- --- --- --- --- --- --- --- --- ---
GFP-19  --- --- --- --- --- --- --- --- --- --- --- --- --- --- --- ---
```

Supplementary Figure 1A
Cummings et al.

LacI-GFP-HP1

```
DT40        TCT CCC TCT CCA GGT TCC CTG GTG CAG GCA GCG CTG ACT CAG CCG GCC TCG GTG TCA GCA AAC CTA
HP1-1       --- --- --- --- --- --- --- --- --- --- --- --- --- --- --- --- --- --- --- --- --- ---
HP1-2       --- --- --- --- --- --- --- --- --- --- --- --- --- --- --- --- --- --- --- --- --- ---
HP1-3       --- --- --- --- --- --- --- --- --- --- --- --- --- --- --- --- --- --- --- --- --- ---
HP1-4       --- --- --- --- --- --- --- --- --- --- --- --- --- --- --- --- --- --- --- --- --- ---
HP1-5       --- --- --- --- --- --- --- --- --- --- --- --- --- --- --- --- --- --- --- --- --- ---
HP1-6       --- --- --- --- --- --- --- --- --- --- --- --- --- --- --- --- --- --- --- --- --- ---
HP1-7       --- --- --- --- --- --- --- --- --- --- --- --- --- --- --- --- --- --- --- --- --- ---
HP1-8       --- --- --- --- --- --- --- --- --- --- --- --- --- --- --- --- --- --- --- --- --- ---
HP1-9       --- --- --- --- --- --- --- --- --- --- --- --- --- --- --- --- --- --- --- --- --- ---
HP1-10      --- --- --- --- --- --- --- --- --- --- --- --- --- --- --- --- --- --- --- --- --- ---
HP1-11      --- --- --- --- --- --- --- --- --- --- --- --- --- --- --- --- --- --- --- --- --- ---
HP1-12      --- --- --- --- --- --- --- --- --- --- --- --- --- --- --- --- --- --- --C --- -T- --G
                     T                                         A
HP1-13      --- --- --- --- --- --- --- C-A --T --- --- --- --- --- --- T-- --- --A --T --- --- ---
HP1-14      --- --- --- --- --- --- --- --- --- --- --- --- --- --- --- --- --- --- --- --- --- ---

DT40        GGA GGA ACC GTC AAG ATC ACC TGC TCC GGG GGT GGC AGC TAT GCT GGA AGT TAC TAT TAT GGC TGG
HP1-1       --- --- --- --- --- --- --- --- --- --- --- --- --- --- --- --- --- --- --- --- --- ---
HP1-2       --- --- --- --- --- --- --- --- --- --- --- --- --- --- --- --- --- --- --- --- --- ---
HP1-3       --- --- --- --- --- --- --- --- --- --- --- --- --- --- --- --- --- --- --- --- --- ---
HP1-4       --- --- --- --- --- --- --- --- --- --- --- --- --- --- --- --- --- --- --- --- --- ---
HP1-5       --- --- --- --- --- --- --- --- --- --- --- --- --- --- --- --- --- --- --- --- --- ---
HP1-6       --- --- --- --- --- --- --- --- --- --- --- --- --- --- --- --- --- --- --- --- --- ---
                                                                   G
HP1-7       --- --- --- --- --- --- --- --- --- --- --- --- --- --- --- --- --- --- --- --- --- ---
HP1-8       --- --- --- --- --- --- --- ---▲--- --- --- --- --- --- --- --- --- --- --- --- --- ---
HP1-9       --- --- --- --- --- --- --- --- --- --- --- --- --- --- --- --- --- --- --- --- --- ---
HP1-10      --- --- --- --- --- --- --- --- --- --- --- --- --- --- --- --- --- --- --- --- --- ---
                                                                   G
HP1-11      --- --- --- --- --- --- --- --- --- --- --- --- --- --- C-- --- --- --- --- --- --- ---
HP1-12      --C -C- --- --- --- --- --- --- --- --- --- --- --- --- --- --- --- --- --- --- --- ---
HP1-13      --- --- --A --- --- --- --A --- --- -A- --- --- --- --- --- --- --- --- --- --- --- ---
                                                                   G
HP1-14      --- --- --- --- --- --- --- --- --- --- --- --- --- --- --- --- --- --- --- --- --- ---

DT40        TAC CAG CAG AAG TCT CCT GGC AGT GCC CCT GTC ACT GTG ATC TAT GAC AAC GAC AAG AGA CCC TCG
HP1-1       --- --- --- --- --- --- --- --- --- --- --- --- --- --- --- --- --- --- --- --- --- ---
HP1-2       --- --- --- --- --- --- --- --- --- --- --- --- --- --- --- --- --- --- --- --- --- ---
HP1-3       -T- --- --- --- --- --- --- --- --- --- --- --- --- --- --- --- --- --- --- --- --- ---
HP1-4       --- --- --- --- --- --- --- --- --- --- --- --- C-- --- --- --- --- --- --- --- --- ---
HP1-5       --- --- --- --- --- --- --- --- --- --- --- --- --- --- --- --- --- --- --- --- --- ---
HP1-6       --- --- --- --- --- --- --- --- --- --- --- --- --- --- --- --- --- --- --- --- --- ---
HP1-7       --- --- --- --- --- --- --- --- --- --- --- --- --- --- --- --- --- --- --- --- --- ---
HP1-8       --- --- --- --- --- --- --- --- --- --- --- --- --- --- --- --- --- --- --- --- --- ---
HP1-9       --- --- --- --- ---▲--- --- --- --- --- --- --- --- --- --- --- --- --- --- --- --- ---
HP1-10      --- --- --- --- --- --- --- --- --- --- --- --- --- --- --- --- --- --- --- --- --- ---
HP1-11      --- --- --- --- --- --- --- --- --- --- --- --- C-- --- --- C-- --- C-- --- --- --- ---
HP1-12      --- --- --- --- --- --- --- --- --- --- --- --- --- --- --- --- --- -C- --- --- --- ---
                                                                               G
HP1-13      --- --- --- --- --- --- --- --- --- --- --- --- --- --- --- --- --- --- --- --- --- ---
HP1-14      --- --- --- --- --- --- --- --- --- --- --- --- --- --- --- --- --- --- --- --- --- ---

DT40        GAC ATC CCT TCA CGA TTC TCC GGT TCC CTA TCC GGC TCC ACA AAC ACA TTA ACC ATC ACT GGG GTC
HP1-1       --- --- --- --- --- --- --- --- --- --- --- --- --- --- --- --- --- --- --- --- --- ---
HP1-2       --- --- --- --- --- --- --- --- --- --- --- --- --- --- --- --- --- --- --- --- --- ---
HP1-3       --- --- --- --- --- --- --- --- --- --- --- --- --- --- --- --- --- --- --- --- --- ---
HP1-4       --- --- --- --- --- --- --- --- --- --- --- --- --- --- --- --- --- --- --- --- --- ---
HP1-5       --- --- --- --- --- --- --- --- --- --- --- --- --- --- --- --- --- --- --- --- --- ---
HP1-6       --- --- --- --- --- --- --- --- --- --- --- --- --- --- --- --- --- --- --- --- --- ---
HP1-7       --- --- --- --- --- --- --- --- --- --- --- --- --- --- --- --- --- --- --- --- --- ---
HP1-8       --- --- --- --- --- --- --- --- --- --- --- --- --- --- --- --- --- --- --- --- --- ---
HP1-9       --- --- --- --- --- --- --- --- --- --- --- --- --- --- --- --- --- --- --- --- --- ---
HP1-10      --- --- --- --- --- --- --- --- --- --- --- --- --- --- --- --- --- --- --- --- --- ---
HP1-11      --- --- --- --- --- --- --- --- --- --- --- --- --- --- --- --- --- --- --- --- --- ---
HP1-12      --- --- --- --- --- --- --- --- --- --- --- --- --- --- --- --- --- --- --- --- --- ---
HP1-13      --- --- --- --- --- --- --- --- --- --- --- --- --- --- --- --- --- --- --- --- --- ---
HP1-14      --- --- --- --- --- --- --- --- --- --- --- -T- --- --- --- --- --- --- --- --- --- ---
```

```
DT40     CGA GCC GAT GAC GAG GCT GTC TAT TTC TGT GGG AGT GCA GAC AAC AGT GGT GCT GCA TTT GGG GCC
HP1-1    --- --- --- --- --- --- --- --- --- --- --- --- --- --- --- --- --- --- --- --- --- ---
HP1-2    --- --- --- --- --- --- --- --- --- --- --- --- --- --- --- --- --- --- --- --- --- ---
HP1-3    --- --- --- --- --- --- --- --- --- --- --- --- --- --- --- --- --- --- --- --- --- ---
HP1-4    --- --- --- --- --- --- --- --- --- --- --- --- --- --- --- --- --- --- --- --- --- ---
HP1-5    --- --- --- --- --- --- --- --- --- --- --- --- --- --- --- --- --- --- --- --- --- ---
HP1-6    --- --- --- --- --- --- --- --- --- --- --- -C- --- --- --- --- --- --- --- --- --- ---
HP1-7    --- --- --- --- --- --- --- --- --- --- --- --- --- --- --- --- --- --- --- --- --- ---
HP1-8    --- --- --- --- --- --- --- --- --- --- --- --- --- --- --- --- --- --- --- --- --- ---
HP1-9    --- --- --- --- --- --- --- --- --- --- --- --- --- --- --- --- --- --- --- --- --- ---
HP1-10   --- --- --- --- --- --- --- --- --- --- --- --- --- --- --- --- --- --- --- --- --- ---
HP1-11   --- --- --- --- --- --- --- --- --- --- --- --- --- --- --- --- --- --- --- --- --- ---
HP1-12   --- --- --- --- --- --- --- --- --- --- --- --- --- --- --- --- --- --- --- --- --- ---
HP1-13   --- --- --- --- --- --- --- --- --- --- --- --- --- --- --- --- --- --- --- --- --- ---
HP1-14   --- --- --- --- --- --- --- --- --- --- --- --- --- --- --- --- --- --- --- --- --- ---

DT40     GGG ACA ACC CTG ACC GTC CTA GGT GAG TCG CTG ACC TCG TCT CGG TCT
HP1-1    --- --- --- --- --- --- --- --- --- --- -A- --- --- --- --- ---
HP1-2    --- --- --- --- --- --- --- --- --- --- -G- --- --- --- --- ---
HP1-3    --- --- --- --- --- --- --- --- --- --- --- --- --- --- --- ---
HP1-4    --- --- --- --- --- --- --- --- --- --- --- --- --- --- --- ---
HP1-5    --- --- --- --- --- --- --- --- --- --- --- --- --- --- -A- ---
HP1-6    --- --- --- --- --- --- --- --- --- --- --- --- --- --- --- ---
HP1-7    --- --- --- --- --- --- --- --- --- --- --- --- --- --- --- ---
HP1-8    --- --- --- --- --- --- --- --- --- --- --- --- --- --- --- ---
HP1-9    --- --- --- --- --- --- --- --- --- --- --- --- --- --- --- ---
HP1-10   --- --- --- --- --- --- --- --- --- --- -▲  -T- --- --- --- ---
HP1-11   --- --- --- --- --- --- --- --- --- --- --- --- --- --- --- ---
                  C
HP1-12   --- --- --- --- --- --- --- --- --- --- --- --- --- --- --- ---
HP1-13   --- --- --- --- --- --- --- --- --- --- --- --- --- --- --- ---
HP1-14   --- --- --- --- --- --- --- --- --- --- --- --- --- --- --- ---
```

Supplementary Figure 1A
Cummings et al.
